# Challenges in the diagnosis and management of Pseudohypoaldosteronism Type 1

**DOI:** 10.1186/1687-9856-2015-S1-P126

**Published:** 2015-04-28

**Authors:** Uma Visser, Paul Jenkins, Tony Lafferty

**Affiliations:** 1 Department of Paediatrics, The Canberra Hospital, Canberra, ACT, Australia; 2 Australian National University, Canberra, ACT, Australia

## 

Autosomal recessive Pseudohypoaldosteronism Type I (PHA-I, MIM#264350), is a rare disease with a severe clinical phenotype [1,2] and generally no improvement with age [3]. It results from mutations in the amiloride-sensitive epithelial sodium channel causing mineralocorticoid-resistant (ENaC), systemic salt wasting, and is lethal without ongoing supra-physiological sodium supplementation and management of hyperkalaemia [4,5].

Other manifestations of systemic PHA-I include decreased sodium-dependant clearance of alveolar fluid causing recurrent chest congestion, cough and wheeze, but no airway infection by bacterial pathogens typifying cystic fibrosis[6,7]; and skin rashes from inflammation of sodium-blocked sweat glands [8] with recurrent Staphylococcal skin infections described [9].

Our patient, now nearly four years old, presented as a day 6 neonate with vomiting, apnoea and floppiness. She was shocked and dehydrated with hyponatraemia, marked hyperkalaemia with runs of ventricular tachycardia.

PHA-1 was diagnosed with high cortisol, renin and aldosterone, normal 17-hydroxyprogesterone, inappropriately high spot urine sodium with low potassium, and normal renal ultrasound. Diagnosis was genetically confirmed with the finding of two inherited, distinct, disease causing mutations in the SCNNIA gene. Increased sweat sodium confirmed systemic salt-wasting. Initial management included a high-sodium, low-potassium formula (Kindergen) supplemented with 22 mmol/Kg/day of enteral sodium, the use of daily potassium binding resin (Resonium), and trial of Fludrocortisone.

Requirement for daily large doses of sodium 16 mmol/Kg/day, provided as a mix of sodium citrate and sodium chloride, and daily Resonium continues. Significant oral aversion remains an issue with good growth achieved on calorie-concentrated Kindergen. Gastrostomy feeding was changed to percutaneous jejunal continual feeding due to persistent gastric emptying problems. Central venous access established early has posed ongoing challenges, with intermittent Staphylococcal infections. Our patient has also been hypertensive requiring medical management from 6 months age. She has a recurrent moist cough with past Haemophilus infection.

Minimising inpatient management is vital for establishing 'normality' and optimising development. The need for frequent electrolyte monitoring and adjustment of sodium intake is managed creatively between home and hospital in close liaison with her paediatric endocrinologist and community-based supports. Clear emergency management plans involving early symptom recognition and rapid hospital-access are instituted to manage salt-wasting episodes.

We shall discuss the challenges and pitfalls of managing this rare, life-threatening disease with sparse long-term prognostic information, in the Australian health care context.

Written informed consent was obtained from the parent of the patient for publication of this abstract and any accompanying images. A copy of the written consent is available for review by the Editor of this journal.

## References

[B1] Riepe FG Clinical and molecular features of type 1 pseudohypoaldosteronism Horm Res 2009 72 1 9 19571553 10.1159/000224334

[B2] Zennaro MC Lombes M Mineralocorticoid resistance Trends Endocrinol Metab 2004 15 264 270 10.1016/j.tem.2004.06.003 15358279

[B3] Adachi M Asakura Y Muroya K Tajima T Fujieda K Kuribayashi E Uchida S Increased Na reabsorption via the Na-Cl cotransporter in autosomal recessive pseudohypoaldosteronism Clin Exp Nephrol 2010 14 228 232 10.1007/s10157-010-0277-0 20376516

[B4] Furgeson SB Linas S Mechanisms of type I and type II pseudohypoaldosteronism J Am Soc Nephrol 2010 21 1842 5 10.1681/ASN.2010050457 20829405

[B5] Güran T Deirmenci S Bulut YK Say A Riepe FG Giiran Ö Critical points in the management of pseudohypoaldosteronism type I J Clin Res Pediatr Endocrinol 2011 3 98 100 10.4274/jcrpe.v3i2.20 21750640 PMC3119449

[B6] Kerem E Bistritzer T Hanukoglu A Pulmonary epithelial sodium-channel dysfunction and excess airway liquid in pseudohypoaldosteronism N Engl J Med 1999 341 156 162 10.1056/NEJM199907153410304 10403853

[B7] Eaton DC Helms MN Koval M The contribution of epithelial sodium channels to alveolar function in health and disease Annu Rev Physiol 2009 71 403 23 10.1146/annurev.physiol.010908.163250 18831683

[B8] Urbatsch A Palier AS Pustular miliaria rubra: a specific cutaneous finding of type I pseudohypoaldosteronism Pediatr Dermatol 2002 19 317 9 10.1046/j.1525-1470.2002.00090.x 12220275

[B9] Saravanapandian N Paul S Matthai J Pseudohypoaldosteronism Type 1: A Rare Cause of Severe Dyselectrolytemia and Cardiovascular Collapse in Neonates J Clin Neonatol 2012 1 224 6 10.4103/2249-4847.106007 24027733 PMC3762049

